# Use of a RT-qPCR Method to Estimate Mycorrhization Intensity and Symbiosis Vitality in Grapevine Plants Inoculated with *Rhizophagus irregularis*

**DOI:** 10.3390/plants11233237

**Published:** 2022-11-25

**Authors:** Morgane Duret, Xi Zhan, Lorène Belval, Christine Le Jeune, Réjane Hussenet, Hélène Laloue, Christophe Bertsch, Julie Chong, Laurence Deglène-Benbrahim, Laure Valat

**Affiliations:** 1 Laboratoire Vigne, Biotechnologies et Environnement, Université de Haute Alsace, Université de Strasbourg, E.A. 3991, 33 rue de Herrlisheim, BP 50568, CEDEX 008, 68000 Colmar, France; 2 Département Génie Biologique, Institut Universitaire de Technologie, 29 rue de Herrlisheim, BP 50568, CEDEX 008, 68000 Colmar, France

**Keywords:** arbuscular mycorrhizal fungus, *Rhizophagus irregularis*, grapevine, controlled conditions, RT-qPCR, symbiosis assessment

## Abstract

Assessing the mycorrhization level in plant roots is essential to study the effect of arbuscular mycorrhizal fungi (AMF) on plant physiological responses. Common methods used to quantify the mycorrhization of roots are based on microscopic visualization of stained fungal structures within the cortical cells. While this method is readily accessible, it remains time-consuming and does not allow checking of the symbiosis vitality. The aim of this work is thus to develop an efficient method for assessing the intensity and vitality of mycorrhiza associated with grapevine through gene expression analyses by RT-qPCR. To this end, grapevine plants were inoculated with the AMF *Rhizophagus irregularis* (Ri). The relationship between mycorrhization level, assessed by microscopy, and expression of several fungus and grapevine genes involved in the symbiosis was investigated. In AMF-inoculated plants, transcript amounts of fungal constitutively-expressed genes *Ri18S*, *RiTEF1α* and *RiαTub* were significantly correlated to mycorrhization intensity, particularly *Ri18S*. Grapevine (*VvPht1.1* and *VvPht1.2*) and AMF (*GintPT*, *Ri14-3-3* and *RiCRN1*) genes, known to be specifically expressed during the mycorrhizal process, were significantly correlated to arbuscular level in the whole root system determined by microscopy. The best correlations were obtained with *GintPT* on the fungal side and *VvPht1.2* on the plant side. Despite some minor discrepancies between microscopic and molecular techniques, the monitoring of *Ri18S*, *GintPT* and *VvPht1.2* gene expression could be a rapid, robust and reliable method to evaluate the level of mycorrhization and to assess the vitality of AMF. It appears particularly useful to identify AMF-inoculated plants with very low colonization level, or with non-active fungal structures. Moreover, it can be implemented simultaneously with the expression analysis of other genes of interest, saving time compared to microscopic analyses.

## 1. Introduction

Grapevine is of great economic importance worldwide. Unfortunately, main cultivated varieties are susceptible to serious diseases. Mycorrhization by arbuscular mycorrhizal fungi (AMF) is known to have positive effects on grapevine growth and resistance to biotic and abiotic stresses [1,2,3]. In this context, the use of AMF as a biological amendment is currently considered as a promising way to improve the growth and resistances of grapevine. In order to better understand the mechanisms related to these beneficial effects, current studies focus on the physiological responses of grapevine to mycorrhizal inoculation with AMF. These studies on different grape variety/AMF strain combinations could then be facilitated by the use of a simple culture model enabling a fine control of plant growth conditions and AMF inoculation. This model, developed previously [4], is well suited to short-term mycorrhization studies and reproducible. The use of an inert substrate and nutrient solutions is also an ideal way to monitor plant nutrition, and intact root systems can be easily recovered from the substrate. Moreover, the resulting root material contains fungal associations of known age, an important parameter for the analysis of plant responses and for an accurate estimation of the mycorrhization level [5]. The assessment of (i) the level of root colonization by AMF and (ii) the vitality of symbiosis also appears as key components of these studies. Methods based on the staining of plant roots and their observation using traditional microscopic observations are the most frequently used [5,6]. Based on the detection of AMF-associated structures within the cortical cells of roots, these methods allow to evaluate various parameters related to the level of mycorrhization [6]. However, this quantification method remains rather subjective, with an interpretation depending on training/experience of operator, and is also time-consuming because of the manual counting of stained AMF structures [7,8,9]. Moreover, the non-vital staining techniques make it difficult to distinguish an active symbiosis from a non-active one [5,7,10]. A molecular method based on an RT-qPCR approach appears promising to assess both the level of mycorrhization and the vitality of the symbiosis and could also be combined with expression analyses of genes of interest. This RT-qPCR technique is known to be more specific to AMF than approaches based on the lipid markers because the presence of the same fatty acids in other microbes confounds the results [9]. Moreover, it allows a better assessment of mycorrhization intensity, unlike classical qPCR, which is influenced by the gradual process of AMF colonization and changes in their nucleic acid content, especially due to the accumulation of structures with a high density of nuclei, such as intraradical spores of some species [7,11,12,13]. In fact, in several studies, expression of AMF reference genes such as Translation Elongation Factor 1α [14,15,16,17] and α-tubulin [18,19,20] have already been used successfully as an indicator of fungal biomass. Furthermore, the use of RT-qPCR allows the distinction between active and non-active fungal structures [12] and thus, the evaluation of the symbiosis functionality. Indeed, expression of genes directly related to a key feature of mycorrhizal symbiosis suggests active symbiosis [11]. In this case, the quantification of target gene transcripts, from the host plant and/or AMF, specifically induced during mycorrhizal colonization, can be monitored. On the plant side, the most common genes used are the AMF-induced phosphate transporter genes, e.g., PT4 in *Medicago truncatula*, *Lycopersicon esculentum* and *Solanum tuberosum* [11,21,22], and PT11 in *Oryza sativa* [23]. They are reliable plant molecular markers of a functional AMF symbiosis [24]. In a previous work on grapevine, we also demonstrated that the presence of many arbuscules in the cortex was associated with a high-level expression of two phosphate transporter genes (PHT1) in roots [4]. These two PHT1 genes are thus good candidates as markers of symbiosis vitality in grapevine. On the AMF side, our study focused on *Rhizophagus irregularis* (formerly *Glomus intraradices*) because published genotype facilitates identification of genes of interest. Many fungal genes also known to be involved in symbiosis [25] could be used to estimate the symbiosis vitality, e.g., phosphate transporters [26], monosaccharide transporters [14], copper transporters [16], ammonium transporters [27], crinkler effector [15], superoxide dismutases [28] or 14-3-3-like proteins [29]. The root colonization by AMF follows a series of different steps (for review: [30,31]) and choosing genes representative of these stages may be relevant. To our knowledge, only one study reported AMF assessment by molecular methods in grapevine, showing a strong correlation between mycorrhization level and fungal DNA concentration in roots, except for old symbiosis [10].

This study aims to overcome the limitations of the usual staining method by developing an alternative and/or complementary molecular technique based on RT-qPCR to assess both the level of mycorrhization and the vitality of the symbiosis in grapevine. For this work, we selected fungal reference genes: *RiTEF1α* (Translation Elongation Factor 1α), *RiαTub* (α-tubulin) and *Ri18S* (component of the small eukaryotic ribosomal subunit 40S). We also chose fungal genes involved in different steps of the symbiosis: *GintPT* (phosphate transporter) and *Ri14-3-3* (encoding a 14-3-3 protein subunit), both expressed from the rhizodermal penetration to the step of arbuscular fine branch formation [26,29], and *RiCRN1* (crinkler effector), expressed from the arbuscular trunk formation to the arbuscules degeneration [15]. Grapevine PHT1 genes (*VvPht1.1* and *VvPht1.2*), whose expression is stimulated during the mycorrhization process [4] were also studied. We then investigated the relationship between quantitative analysis of the expression of plant- and AMF-associated genes by RT-qPCR and the level of mycorrhizal colonization by microscopic observation of stained AMF structures.

## 2. Methods

### 2.1. Plant Material

Plantlets of Syrah (*Vitis vinifera* cv. Mondeuse blanche B x Duzera N.) were propagated in vitro by single-node cuttings and transferred to individual pots filled with sterilized sand/perlite mix (1:1) for ex vitro acclimatization. They were cultivated as described in [4]. Briefly, growth was carried out in a climatized chamber with a 16 h photoperiod (150 μEm^−2^s^−1^ light irradiance), at 24/20 °C (day/night) and 60% relative humidity. Plants were watered to saturation twice a week with a complete nutrient solution containing 2.5 mM Ca(NO_3_)_2_; 2.5 mM KNO_3_; 0.5 mM KH_2_PO_4_; 1 mM MgSO_4_; 50 µM EDTA-Fe(III)-Na; 10 µM H_3_BO_3_; 2 µM MnCl_2_; 1 µM ZnSO_4_; 0.5 µM CuSO_4_ and 0.05 µM Na_2_MoO_4_. One week before *Rhizophagus irregularis* inoculation, the plants were watered with the same nutrient solution, except for KH_2_PO_4_ lowered to 0.1 mM (low-Pi).

### 2.2. Mycorrhizal Inoculum and Inoculation

The AM fungal inoculum was *Rhizophagus irregularis* DAOM197198 (Agronutrition, Carbonne, France). Ninety-five plants were inoculated with 1000 spores in 20 mL aqueous suspension poured at the base of the plant stem. As control, a batch of plants received the same volume of sterile demineralized water (non-inoculated plants). Plants were watered with tap water for 1 week, then with the low-Pi nutrient solution for 5 additional weeks. All analyses were performed on the AMF-inoculated and the control plants 6 weeks after the inoculation.

### 2.3. Root Staining and Assessment of AMF Colonization by Microscopy

After harvest, the young and non-lignified roots were randomly sampled from each plant and stained using a slightly modified ink and vinegar protocol based on Vierheilig [32]. Briefly, the roots (approximately 1 g) were cleared in 5 mL 10% KOH for 20 min at 90 °C, and treated with 5 drops of 30% H_2_O_2_. They were rapidly rinsed three times with demineralized water and stained for 5 min in 5% black Sheaffer ink and 8% acetic acid at 90 °C. The roots were then rinsed three times with demineralized water and destained for 15 min in 8% acetic acid at room temperature. Samples were finally stored in lactoglycerol (1:1:1 lactic acid/glycerol/water) at 4 °C. Thirty root segments (each 1 cm long) were randomly collected from each sample, mounted 10 per slide in glycerol and observed at 100× magnification. Based on the Trouvelot method [33] and on the Mycocalc program (https://www2.dijon.inrae.fr/mychintec/Mycocalc-prg/download.html (downloaded on 15 May 2019), the following parameters related to the AMF colonization were calculated: ‘M%’ as an assessment of the proportion of fungal structures in the root cortex (by its calculation method, M% reflects both mycorrhization frequency and intensity), and ‘A%’ as arbuscule abundance in the whole root system. Briefly, through microscopic observation, two scores were assigned to each of the thirty stained root fragments: one to rate mycorrhization intensity (from 0 to 5) and the other to rate the arbuscular abundance (from A0 to A3). M% is then calculated: (95n5 + 70n4 + 30n3 + 5n2 + n1)/(number of total fragments) where n5 = number of fragments rated 5 according to mycorrhizal colonization; n4 = number of fragments rated 4, etc. A% is calculated from M% and a% (absolute arbuscule richness) as A% = a × M/100. a% = (100mA3 + 50mA2 + 10mA1)/100 where mA3, mA2 and mA1 are the % of m, rated A3, A2 and A1, respectively, with mA3 = ((95n5A3 + 70n4A3 + 30n3A3 + 5n2A3 + n1A3)/number of mycorrhized fragments) × 100/m and the same for A2 and A1, and with m% = M% × (number of total fragments)/(number of mycorrhized fragments), as previously described in [6]. For this study, 40 plants were chosen among 95 in order to obtain a homogeneous distribution of the plants over a wide range of mycorrhization intensity and arbuscule abundance, and to avoid bias in the calculation of the correlation coefficient. Five non-inoculated plants were also stained to check the absence of mycorrhization.

### 2.4. RNA Extraction from Roots

Simultaneously, 65–75 mg of fresh, young and non-lignified roots were collected from each plant and stored at −80 °C. Total RNA was extracted using the Direct-zolTM RNA kit (Zymo Research, Irvine, California, USA), except for the first step that was performed with the ConcertTM Plant RNA Reagent InVitrogen (Thermo Fisher Scientific, Waltham, Massachusetts, USA) after addition of PVP-40 (3% m/v final). Moreover, DNase I treatment was performed during RNA extraction (15 min, at room temperature), according to the manufacturer’s protocol. The RNA quantification was carried out on a BioSpec Nano spectrophotometer (Shimadzu Biotech, Marne-la-Vallée, France). Its purity was controlled by 260/280 OD ratio and 260/230 OD ratio, which were between 1.8 and 2. Then, its quality/integrity was checked by agarose gel electrophoresis. RNA was stored in aliquots at −80 °C until use.

### 2.5. Reverse Transcriptase-Quantitative Real-Time PCR

Reverse transcription was performed on 500 ng of RNA with the iScriptTM Reverse Transcription Supermix^®^ (Bio-Rad, Marnes-la-Coquette, France) in Bio-Rad C1000 Thermal Cycler (25 °C, 5 min; 46 °C, 20 min; 95 °C, 1 min; 12 °C, ∞). cDNA was stored in aliquots at −20 °C until use. End-point and quantitative RT-PCR were performed using the conditions described in Supplementary Table S1a,b in Mastercycler Eppendorf and CFX96 Real-Time System (Bio-Rad), respectively. The primers (Table 1) were synthesized from Eurofins Genomics, Ebersberg, Germany. The amplicons obtained by end-point PCR were checked by gel electrophoresis, sequenced (Genoscreen, Lille, France) and aligned against published sequences. The efficiency of each pair of primers was estimated from standard calibration curves based on serial 6-fold dilutions of purified amplicons (10^7^–10^2^ copies). For each sequence, Cq values were plotted against the log_10_ of their copy numbers. The amplification efficiency (E) (Table 1) was estimated using the slope of the standard curve according to the following formula: E = (10^−1/slope^) − 1. For each plant, one RT-qPCR analysis was carried out with the 11 primer pairs with technical triplicates. ‘No-template’ and ‘no RT’ controls have never showed any amplification. The specificity of each PCR amplification procedure was checked with a heat dissociation protocol (from 70 to 90 °C) after the final cycle of the qPCR. The Bio-Rad CFX Maestro inter-run calibration was applied to normalize between the qPCR runs. Cq values of grapevine reference genes, *VvAct* (XM_002282480.2), *Vv60SRP* (XM_002270599.1) and *VvEF1α* (CB977561), were stable whatever the mycorrhization status. The geometric mean of these plant reference genes was used for data normalization. Fungal gene expression was normalized with these plant reference gene expressions as already described [9,16]. Target gene expression in AMF-inoculated plants was quantified relatively to the mean expression of 5 non-inoculated controls (Pfaffl 2001 method [34]). When the target gene was not expressed in controls, Cq value of 38 was used to calculate ΔCt. When the target gene was not expressed in inoculated plants, the relative expression was considered as null. The formula used for calculation of relative expression (R) was:

$$R=\frac{E^{\Delta \mathrm{Ct}\;\mathrm{Target}\;\mathrm{gene}}}{\sqrt[{3}]{\left(E^{\left(\Delta \mathrm{Ct}\;\mathrm{Vvact}\right)}\times \;E^{\left(\Delta \mathrm{Ct}\;\mathrm{Vv}60\mathrm{SRP}\right)}\times \;E^{\left(\Delta \mathrm{Ct}\;\mathrm{VvEF}1\alpha \right)}\right)}}$$

with E = PCR efficiency.

### 2.6. Statistical Analysis

Data analyses were performed using R software (V4.0.0). Spearman’s rank correlations were performed between the various parameters related to the level of colonization and the expression of the various genes. The pairwise two-sided *p*-values, adjusted by Holm’s method, were determined to evaluate the significance level of each correlation coefficient. Ascending Hierarchical Classifications (AHC) were performed using the Ward method on standardized data to cluster the inoculated plants according to both their mycorrhizal colonization and gene expression.

## 3. Results

### 3.1. Root Colonization by R. irregularis

No trace of fungal structures was observed in the roots of non-inoculated plants. For this study, 40 plants were chosen according to their level of mycorrhization intensity (M% ranging from 0.03% to 90.33%) and arbuscule abundance (A% ranging from 0 to 64.13%), in order to cover a wide range of colonization levels as potentially obtained in our system.

### 3.2. Correlation between Mycorrhization Intensity and Expression of AMF Constitutive Genes

**Figure 1:**
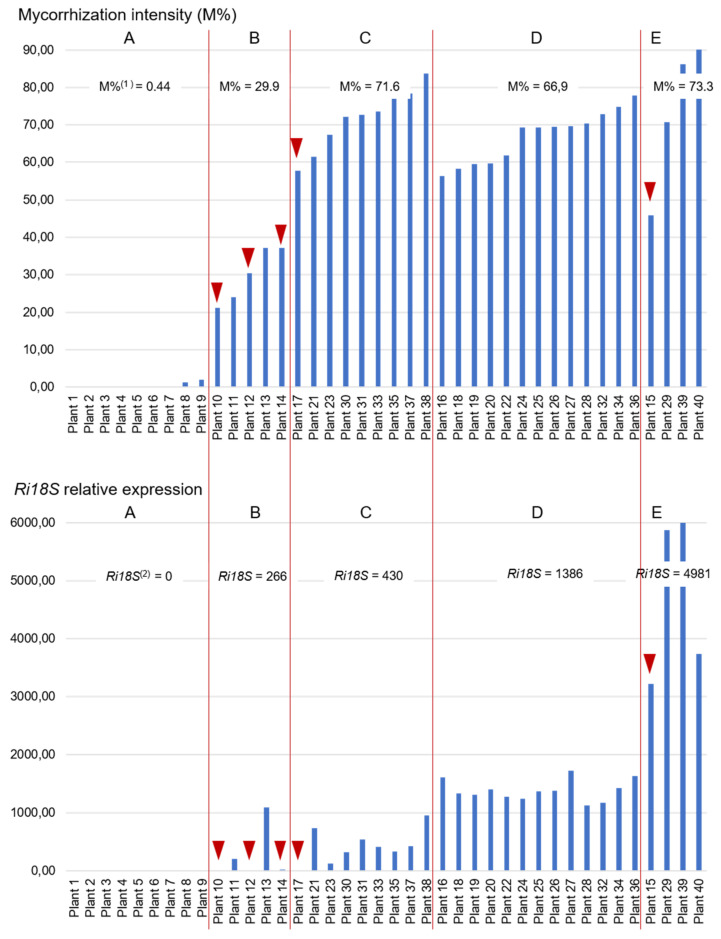
Mycorrhization intensity and relative expression of *Ri18S* gene in 40 grapevine plants. A, B, C, D, E: clusters obtained by Ascending Hierarchical Classification (AHC) on AMF-inoculated plants according to their mycorrhization intensity and *Ri18S* expression; red triangles point to outstanding plants. ^(1)^: mean of mycorrhization intensity of the cluster. ^(2)^: mean of *Ri18S* gene relative expression of the cluster.

We first assessed the relationship between the constitutively expressed *Rhizophagus irregularis* genes *Ri18S*, *RiTEF1α* and *RiαTub* and the mycorrhization intensity (M%). Expression of these genes was not detectable in the roots of non-inoculated control plants. In inoculated-AMF plants, the best correlation was obtained for *Ri18S*, however, the expression of these three genes was significantly correlated with M% (Table 2). For further investigation, M% and relative expression of *Ri18S* were represented for each plant (Figure 1). According to the AHC performed on the mycorrhizal samples based on Ri colonization intensity and *Ri18S* relative gene expression, 5 clusters were identified from the dendrogram (Supplementary Figure S1). Plants belonging to group A were very poorly colonized by the AMF and *Ri18S* expression was not detectable. Within the group B, plants had a medium M% (around 30%) and a detectable, but rather low, level of *Ri18S* expression. Strong disparities were noticed among plants, with relative expression ranging from 1.59 to 1090 (Figure 1). Plants of groups C, D and E were quite similar regarding the intensity of mycorrhization (around 70%). However, they differed on the *Ri18S* expression, which was moderate in group C, high in group D and very high in group E, where the average relative expression was more than 3-fold higher than in group D and almost 12-fold higher than in group C. Some plants were peculiar, such as plant 17 in which *Ri18S* was only slightly expressed despite a quite high mycorrhization intensity level. On the contrary, plant 15 had a very high amount of *Ri18S* transcripts, whereas it was moderately colonized by the AMF.

### 3.3. Correlation between Arbuscular Rate and Gene Expression

Second, we evaluated both plant (*VvPht1.1* and *VvPht1.2*) and fungal (*GintPT*, *Ri14-3-3* and *RiCRN1*) genes, well-expressed in the different symbiosis stages, and determined their relationship with the arbuscule abundance in the whole root system (A%). This parameter is indeed considered as the best indicator of mycorrhiza vitality [33,35]. In the roots of the control plants, expression of *GintPT*, *Ri-14-3-3*, *RiCRN1* and *VvPht1.1* was not detectable, whereas *VvPht1.2* transcripts were detected at very low levels as it was also observed in a previous study [4]. In the roots of mycorrhizal plants, best correlation with A% was obtained with *GintPT* followed by *Ri14-3-3* and *RiCRN1* relative expression, and all were significant (Table 3). Regarding grapevine genes, *VvPht1.1* and *VvPht1.2* were also significantly correlated with A%, though less than the fungal genes (Table 3). *GintPT* and *VvPht1.2* expression being the best related to arbuscular abundance in Ri and grapevine, respectively, they were therefore selected as putative markers of mycorrhiza vitality. Interestingly, *GintPT* and *Vvpht1.2* were also well correlated with M% (respectively ρ = 0.7 and 0.65, significant at *p* < 1%). Plants were then classified by an AHC according to A% and *GintPT* expression on the one hand, and A% and *VvPht1.2* expression on the other hand.

**Figure 2:**
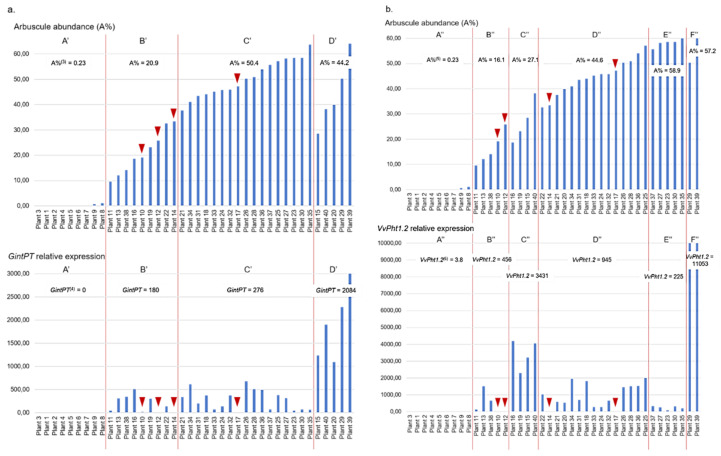
(**a**) Arbuscule abundance and relative expression of *GintPT* gene in 40 grapevine plants. A’, B’, C’, D’: clusters obtained by AHC on AMF-inoculated plants according to their arbuscule abundance and *GintPT* expression ^(3)^: mean of arbuscule abundance of the cluster. ^(4)^: mean of *GintPT* gene relative expression of the cluster. (**b**) Arbuscule abundance and relative expression of *VvPht1.2* gene in 40 grapevine plants. A’’, B’’, C’’, D’’, E’’, F’’: clusters obtained by AHC on AMF-inoculated plants according to their arbuscule abundance and *VvPht1.2* expression. ^(5)^: mean of arbuscule abundance of the cluster. ^(6)^: mean of *VvPht1.2* gene relative expression of the cluster. Red triangles point to outstanding plants.

According to *GintPT* expression and A% dendrogram, the plants could be clustered in 4 groups (Supplementary Figure S2a). Again, group A’ consisted of the same plants as previously described, with very low arbuscule abundance and with no detectable *GintPT* expression (Figure 2a). Plants of group B’ displayed a moderate arbuscule abundance (around 20%) along with a medium *GintPT* expression level. In group C’, A% levels were important (50% on average) and *GintPT* expression was fairly high. Group D’ was made up of plants with a slightly lower arbuscule abundance (44% on average) but with *GintPT* expression almost 8 times higher than in Group C’ (Figure 2a).

Regarding *VvPht1.2* expression and A% dendrogram, plants were divided into 6 groups (Supplementary Figure S2b). Group A” consisted of the same plants as in previous classifications, characterized by very low arbuscule level and very weak *VvPht1.2* expression (Figure 2b). Plants of group B” had quite low arbuscule abundance, between 10 to 25%. *VvPht1.2* expression was rather important on average, but with a great heterogeneity, ranging from 1.49 to 1509. In group C”, the arbuscules percentage was moderate, but the gene expression was very high. In contrast, group D”, which presented a greater richness in arbuscules, had an average *VvPht1.2* expression level 3.6 times lower. Groups E” and F” were the groups wherein the arbuscule abundance was the greater but with very contrasted results regarding gene expression. Indeed, plants of group E” were characterized by a rather medium relative expression of *VvPht1.2*, 4 times lower than in group D”. In two plants (29 and 39) of group F”, expression of *VvPht1.2* was drastically up-regulated.

**Figure 3:**
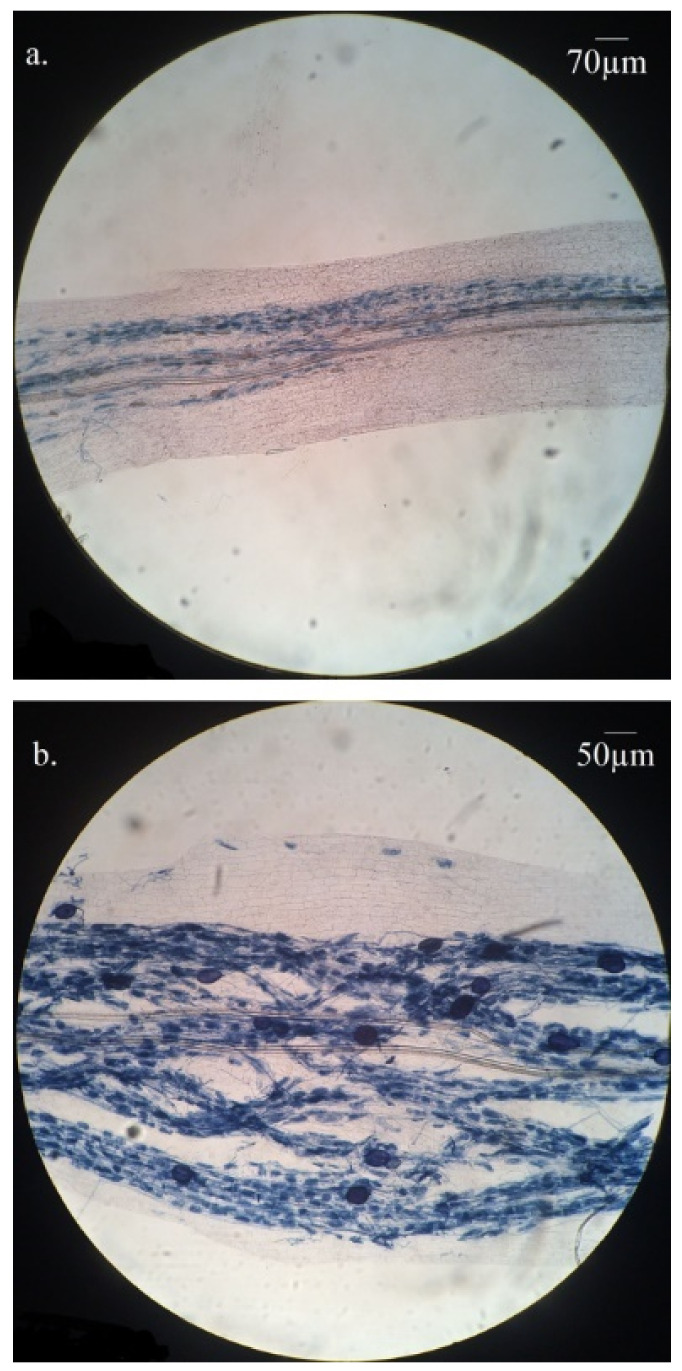
Microscopic observations after black Sheaffer ink staining. (**a**) arbuscules not clearly visible. (**b**) well-shaped arbuscules and many spores.

Some plants also present contradictory results between mycorrhization level assessed by microscopy and gene expression (Figure 1 and Figure 2). On the one hand, some plants were found with both moderate to high mycorrhization intensity and arbuscule abundance, and low *Ri18S*, *GintPT* and *VvPht1.2* expression (plants 12, 14 and 17, for example). In these plants, roots showed few spores, and about half of the observed arbuscules were not clearly visible, as they were probably degenerating (Figure 3a). On the other hand, a few plants presented moderate mycorrhization intensity and high amounts of *Ri18S* transcripts, such as plants 13 and 15, in which well-shaped arbuscules and many spores were observed (Figure 3b).

## 4. Discussion

Significant correlations were obtained between microscopic observations and all fungal and grapevine gene expressions studied, as it was already shown with biomass indicators [16,18] and with plant Pi transporters [11,23], for other plant-AMF combinations. *Ri18S* was the fungal reference gene best correlated with mycorrhization intensity, while the best correlations with arbuscular abundance were obtained with *GintPT* and *VvPht1.2* expression, among studied genes involved in different steps of the symbiosis.

However, some disparities can be noted between groups of plants with close mycorrhization levels but quite different gene expression. These different results may be explained partly because staining requires about 1 g of roots against only 70 mg for RNA extractions, which may not be representative enough. As specified by Gamper et al. [13] and Voříšková et al. [36], mycorrhizae are not uniformly distributed in plant roots and it cannot be excluded that collected samples did not accurately reflect the real mycorrhization plant status. Another reason could be that microscopic evaluations do not distinguish between senescent and active fungal structures, as previously reported [5,6]. Especially, neighboring root cells can host senescent or young arbuscules, as cell recolonization by AMF is a common process [37]. This may explain why, for a strong level of mycorrhizal structures, *Ri18S* expression varied from moderate to very high (Figure 1, groups C, D and E). Contrasted results were also noticed between plants of group C”, with an average arbuscular abundance and a very high *VvPht1.2* expression, and plants of group D”, with more arbuscules but lower level of gene expression (Figure 2b). *VvPht1.2*, due to its homology with other Pi-transporter genes [4], can be considered as a symbiosis vitality marker in grapevine, as already described for *MtPT4* in *Medicago truncatula* [11]. Indeed, in addition to their role in Pi-uptake, phosphate transporters could play a role in the morphogenesis regulation and the lifespan of arbuscules, as well as in the symbiosis maintenance [38]. These results suggest that these genes’ up-regulation by AMF colonization does not depend solely on arbuscule richness, but above all, on their viability, as already mentioned by Kobae and Hata [39].

Atypical behavior of some plants led us to re-evaluate the microscopic observations by focusing on the quality of the arbuscules and the spore observation, which are not normally considered in the Trouvelot method [33]. Plants 13 and 15, for example, with moderate mycorrhization intensity and very high amounts of *Ri18S* transcripts showed well-shaped arbuscules and many spores. It has now been demonstrated that AMF intra-radical spores, due to their high-density nuclei, are a great pool of nucleic acids, much larger than that of hyphae [13]. It could explain why plant roots containing many spores exhibit very high amounts of *Ri18S* RNA. In contrast, plants such as 10, 12, 14 and 17 showed moderate to high levels of mycorrhization intensity and moderate arbuscular rate but very low expression of *Ri18S*, *GintPT* and *VvPht1.2*. Careful microscopic observations of these plants showed degraded arbuscules. This supports the above assumption that gene expression level does not only depend on AMF colonization intensity, nor even arbuscule abundance, but also probably on senescent fungal structure proportion, as already proposed [7,10]. Plants 29 and 39 were also notable because *Ri18S*, *GintPT* and *VvPht1.2* transcripts were particularly abundant. This may be due to high mycorrhization colonization and to the presence of a great number of well-developed arbuscules. Thus, it may be supposed that exchanges between AMF and grapevine were especially active in these plants. Interestingly, *GintPT* and *VvPht1.2* expression is highly correlated with colonization levels and could be involved in Pi-uptake, one of the most important exchange pathways between the AMF and the plant. These genes would constitute a good indicator of the quality of the two partners’ exchanges and thus of the symbiosis vitality. They would thus allow to be closer to the biological reality than simple microscopic observations which only quantify the presence and the abundance of viable and non-viable fungal structures.

According to classifications made on mycorrhization parameters obtained by microscopic method and on tracking marker gene expression, two main groups were obtained: (1) the first group consisted of inoculated and slightly mycorrhized plants where genes were not expressed, and with plants moderately mycorrhized but showing a rather low gene expression (represented by groups A, A’, A” and B, B’, B” of the different figures respectively); (2) the second group was composed of plants with moderate to high mycorrhization and a high or very high gene expression. This rough classification allows the identification of plants close to non-mycorrhized ones, or plants with probably deteriorated or senescent fungal structures. It can reasonably be assumed that AMF colonization level or activity is too low to have a significant impact on the plant physiology. Thus, the molecular method we have developed would allow to exclude from analysis plants with low or moderate mycorrhization levels and low marker gene expression. The second group includes well-mycorrhized plants. Although the expression level of tracked genes was unequal in these plants, it was high enough to conclude that the symbiosis is well-developed and viable, and may have an effect on plant physiology. This molecular tool for assessing mycorrhization by *R. irregularis* in grapevine is relevant enough to be used in laboratory studies, in addition to, or in place of, microscopic observations.

## 5. Conclusions

The expression of AMF constitutive genes was monitored and compared to the mycorrhization intensity. Significant correlations were obtained, especially for *Ri18S*. The expression of AMF and grapevine genes involved in symbiosis was also monitored and compared to the arbuscule abundance in the whole root system. Rather good correlations were obtained, in particular for *GintPT* and *VvPht1.2*. To assess the Ri colonization intensity and the symbiosis vitality, the use of RT-qPCR and the assessment of the fungal genes *Ri18S* and *GintPT* expression, as well as the grapevine gene *VvPht1.2* expression, could be relevant.

The advantage of this method based on gene expression analysis is to directly highlight the symbiosis vitality, leading to saving time and objectivity gain compared to staining technique. Furthermore, expression of mycorrhization marker genes can be analyzed simultaneously with other genes of interest, such as defense genes or genes involved in hydromineral nutrition. This molecular technique should be particularly useful in controlled experiments with a known AMF strain since study of gene expression is specific of the AMF species used. The same type of approach should be carried out with other AMF species and other vine genotypes in order to complete the method to evaluate mycorrhization intensity and symbiosis vitality in grapevine plants.

## Figures and Tables

**Table 1:** Oligonucleotide primers. ^(1)^: GenBank; ^(2)^: Genoscope Vitis.

		Genes	Accession Number	Forward Primer/Reverse Primer Sequences	Amplicon Size (bp)	Amplification Efficiency	Reference
**Grapevine**	Reference genes	*VvAct*	XM_002282480.2 ^(1)^	TGCTATCCTTCGTCTTGACCTTG/ GGACTTCTGGACAACGGAATCTC	263	85.8	Reid et al. 2006
		*Vv60SRP*	XM_002270599.1 ^(1)^	TCCATTATTCCCACCTCTCG/ TTGAACTTGCTTCCGGTTCT	213	94.17	Gamm et al. 2011
		*VvEF1α*	CB977561 ^(1)^	AATGGCTATGCCCCTGTTCTG/ CGCCTGTCAATCTTGGTCAGTAT	83	102.04	Reid et al. 2006
	AMF-induced genes expression	*Vvpht1.1*	GSVIVT01028732001 ^(2)^	CAACTTTGTGATTGGGGTTG/ AGAGCAGATGGCACAAATG	136	98.11	Valat et al. 2018
		*Vvpht1.2*	GSVIVT01028733001 ^(2)^	CGTGAGGCGGATTTTCTGT/ ATCAAAGAACTCTCTCGACCAT	246	102.56	Valat et al. 2018
***R. irregularis***	Reference genes	*RiTEF*	XM_025321412.1 ^(1)^	TGTTGCTTTCGTCCCAATATC/ GGTTTATCGGTAGGTCGAG	127	92.71	Manck-Götzenberg et Requena, 2016
		*Riα-tubulin*	XM_025319263.1 ^(1)^	TGTCCAACCGGTTTTAAAGT/ AAAGCACGTTTGGCGTACAT	173	99.13	Watts-William et al. 2017
		*Ri18S*	HE817882.1 ^(1)^	TGTTAATAAAAATCGGTGCGTTGC/ AAAACGC AAATGATCAACCGGAC	451	92.78	Gonzalez-Guerrero et al. 2005
	AMF-induced genes expression	*Ri14.3.3*	AM049264.1 ^(1)^	GCAAGCCGAACGTTATGATG/ GGCAAGGATATCCGAGCATAC	262	93.36	Sun et al. 2018
		*GintPT*	AY037894 ^(1)^	AACACGATGTCAACAAAGCAAC/ AAGACCGATTCCATAAAAAGCA	218	96.76	Fiorilli et al. 2013
		*RiCRN1*	MH542411.1 ^(1)^	GATATAGATAAGGACCAGCTTG/ TGCCAACAGCTCGTCACT	262	96.68	Voβ et al. 2018

**Table 2:** Spearman’s rank correlation coefficients between constitutively expressed *Rhizophagus irregularis* genes and mycorrhization intensity (M%).

	M%	*Ri18S*	*RiTEF1α*	*RiαTub*
**M%**		0.6951 ****	0.6779 ****	0.6688 ****
***Ri18S***			0.9689 ****	0.9702 ****
***RiTEF1α***				0.9706 ****
***RiαTub***				

**** corresponding to levels *p* < 0.0001, pairwise two-sided *p*-values, adjusted by Holm’s method for each rank correlation.

**Table 3:** Spearman’s rank correlation coefficients between expression of *Rhizophagus irregularis* or plant genes and arbuscule abundance in the whole root system (A%).

	A%	*GintPT*	*Ri14-3-3*	*RiCRN1*	*VvPht1.1*	*VvPht1.2*
**A%**		0.5705 **	0.5395 **	0.5366 **	0.4746 *	0.4843 *
***GintPT***			0.9737 ****	0.966 ****	0.9515 ****	0.9159 ****
***Ri14-3-3***				0.9899 ****	0.972 ****	0.9475 ****
***RiCRN1***					0.9609 ****	0.9458 ****
***VvPht1.1***						0.951 ****
***VvPht1.2***						

*, **, and **** corresponding to * *p* < 0.05; ** *p* < 0.01; **** *p* < 0.0001, pairwise two-sided *p*-values, adjusted by Holm’s method for each rank correlation.

## Supplementary Materials

The following supporting information can be downloaded at: https://www.mdpi.com/article/10.3390/plants11233237/s1, Figure S1: Cluster dendrogram obtained by Ascending Hierarchical Classifications (AHC) on 40 grapevine plants according to their mycorrhization intensity and Ri18S expression. AHC were performed using the Ward method on standardized data to cluster inoculated plants according to their mycorrhization intensity and Ri18S expression; Figure S2: Cluster dendrogram obtained by Ascending Hierarchical Classifications (AHC) on 40 grapevine plants according to their arbuscule abundance and fungal or grapevine phosphate transporter gene expression. AHC were performed using the Ward method on standardized data to cluster inoculated plants according to their mycorrhization intensity and Ri18S expression: (a) GintPT; (b) VvPht1.2. Supplementary Table S1: End-point and quantitative RT-PCR conditions (a) end-point PCR and RT-qPCR conditions; (b) amplification program. Supplementary Table S2: Mean Cq and Cq standard deviation (SD) for each plant and each gene.
