# Supplementary material for: Use of a RT-qPCR Method to Estimate Mycorrhization Intensity and Symbiosis Vitality in Grapevine Plants Inoculated with Rhizophagus irregularis
Source: Plants (Basel). 2022 Nov 25;11(23):3237. doi: 10.3390/plants11233237 (PMC9741363; doi:10.3390/plants11233237)
Supplement: Supplementary file 1 [file plants-11-03237-s001.zip › Duret et al, 2022-Supplemental Table S1.pdf]

Table S1. End-point and quantitative RT-PCR conditions (**a**) end-point PCR and RT-qPCR conditions; (**b**) amplification program.

a

		End-point PCR	***RT-qPCR***
solution volume, μL	cDNA template (5ng/μL)	2	5
	forward/reverse primer (10μM)	0.8/0.8	1/1
	dNTPs (5mM)	0.8	0
	$MgCl_2$ (50mM)	0.6	0
	Reaction buffer (10x)	2	0
	Taq polymerase (0.5 u)	0.1	0
	SsoFast™ EvaGreen® Supermix	0	10
	DNase/RNase free water	12.9	3

b

initial denaturation	95° C, 3 min
amplification cycle (40)	95° C, 15 s; 60° C, 15 s; 72° C, 20 s
final extension	72° C, 5 min
