# Supplementary material for: Use of a RT-qPCR Method to Estimate Mycorrhization Intensity and Symbiosis Vitality in Grapevine Plants Inoculated with Rhizophagus irregularis
Source: Plants (Basel). 2022 Nov 25;11(23):3237. doi: 10.3390/plants11233237 (PMC9741363; doi:10.3390/plants11233237)
Supplement: Supplementary file 1 [file plants-11-03237-s001.zip › Duret et al., 2022-Supplemental TableS2.pdf]

Table S2. Mean Cq and Cq standard deviation (SD) for each plant and each gene.

Gene	*VvAct*		*Vv60SRP*		*VvEF1a*		*Ri18S*		*RiTEF1a*		*RiaTub*		*GintPT*		*Ri14-3-3*		*RiCRN1*		*VvPht1.1*		*VvPht1.2*	
**Plant number**	**Mean Cq**	**Cq SD**	**Mean Cq**	**Cq SD**	**Mean Cq**	**Cq SD**	**Mean Cq**	**Cq SD**	**Mean Cq**	**Cq SD**	**Mean Cq**	**Cq SD**	**Mean Cq**	**Cq SD**	**Mean Cq**	**Cq SD**	**Mean Cq**	**Cq SD**	**Mean Cq**	**Cq SD**	**Mean Cq**	**Cq SD**
**1**	19,82	0,04	26,37	0,17	18,78	0,02	40,14	0,00	39,77	0,27	37,92	0,00	38,90	0,00	38,51	0,00	N/A	0,00	38,05	1,16	29,11	0,10
**2**	18,95	0,06	27,04	0,22	19,08	0,20	N/A	0,00	N/A	0,00	N/A	0,00	N/A	0,00	37,63	0,00	N/A	0,00	N/A	0,00	32,98	0,24
**3**	19,21	0,15	27,33	0,33	19,13	0,16	39,38	0,00	N/A	0,00	38,87	0,04	35,74	0,00	N/A	0,00	39,62	0,70	N/A	0,00	32,80	0,17
**4**	20,01	0,09	25,91	0,07	19,18	0,18	40,80	0,73	38,20	0,00	39,09	1,12	N/A	0,00	38,63	0,11	37,85	0,00	38,81	0,12	31,08	0,04
**5**	19,29	0,14	27,05	0,41	18,99	0,15	N/A	0,00	39,26	0,00	N/A	0,00	N/A	0,00	N/A	0,00	N/A	0,00	38,35	0,51	29,75	0,06
**6**	19,53	0,09	26,33	0,10	19,08	0,06	40,44	0,00	38,10	0,00	38,21	0,48	N/A	0,00	38,76	0,33	N/A	0,00	38,07	1,01	30,34	0,14
**7**	18,94	0,19	26,83	0,09	18,63	0,17	N/A	0,00	N/A	0,00	38,12	0,00	38,49	0,00	N/A	0,00	40,06	0,43	37,72	0,96	29,51	0,06
**8**	21,03	0,07	27,31	0,27	19,57	0,07	40,33	0,71	N/A	0,00	40,30	0,46	N/A	0,00	N/A	0,00	N/A	0,00	N/A	0,00	32,41	0,06
**9**	21,24	0,11	26,67	0,15	19,68	0,11	39,87	0,78	38,94	0,00	40,58	0,00	N/A	0,00	39,86	0,00	N/A	0,00	39,16	0,00	33,50	0,10
**10**	22,74	0,17	28,08	0,14	20,98	0,10	34,44	0,19	33,07	0,09	31,52	0,06	35,45	0,27	33,59	0,17	37,61	0,28	34,74	0,25	31,73	0,03
**11**	19,65	0,08	27,82	0,04	19,03	0,04	29,22	0,11	29,21	0,05	27,99	0,09	31,99	0,17	29,89	0,18	33,74	0,25	29,02	0,33	26,06	0,09
**12**	21,13	0,06	27,98	0,18	19,74	0,04	37,56	0,19	37,24	0,23	37,00	0,23	N/A	0,00	37,65	1,90	N/A	0,00	39,04	1,03	32,96	0,22
**13**	18,93	0,12	27,08	0,10	18,79	0,29	26,05	0,09	25,55	0,04	25,13	0,10	28,48	0,20	26,51	0,24	30,60	0,10	25,71	0,15	22,00	0,05
**14**	21,97	0,24	27,15	0,24	19,82	0,27	33,65	0,02	33,45	0,29	31,79	0,48	36,04	0,45	33,65	0,11	35,97	0,21	38,74	0,22	31,23	0,20
**15**	20,75	0,06	27,89	0,15	19,71	0,01	25,58	0,27	26,17	0,08	24,63	0,13	27,56	0,24	25,79	0,14	29,68	0,21	26,33	0,41	22,03	0,17
**16**	20,17	0,08	29,31	0,20	19,98	0,21	27,05	0,24	25,98	0,07	25,77	0,19	29,26	0,18	26,21	0,08	29,95	0,19	26,37	0,41	22,01	0,08
**17**	19,49	0,17	27,51	0,30	19,21	0,31	35,18	0,24	33,48	0,21	33,40	0,23	36,28	0,49	34,27	0,25	37,17	0,27	32,88	0,13	30,68	0,11
**18**	20,91	0,10	27,25	0,20	19,55	0,15	26,72	0,13	27,18	0,15	25,93	0,46	29,12	0,03	27,20	0,04	31,39	0,17	27,08	0,25	22,63	0,02
**19**	20,29	0,03	27,32	0,09	19,21	0,09	26,44	0,14	26,22	0,15	25,16	0,05	29,17	0,25	26,06	0,16	30,14	0,32	26,36	0,19	22,03	0,10
**20**	22,66	0,18	27,00	0,04	19,89	0,09	27,25	0,03	26,06	0,11	24,84	0,09	28,10	0,12	26,30	0,15	30,07	0,09	27,50	0,01	24,96	0,14
**21**	19,17	0,04	27,28	0,08	18,83	0,11	26,82	0,13	26,32	0,04	25,85	0,12	28,50	0,11	27,43	0,04	31,63	0,16	26,88	0,26	23,52	0,11
**22**	20,57	0,16	27,00	0,16	19,10	0,08	26,43	0,17	26,95	0,21	25,72	0,47	30,34	0,24	27,15	0,07	31,22	0,16	27,39	0,08	23,13	0,11
**23**	22,51	0,20	27,65	0,02	20,51	0,04	31,34	0,04	30,90	0,18	29,68	0,11	33,54	0,29	30,85	0,29	35,15	0,64	33,05	0,28	28,07	0,13
**24**	19,81	0,08	28,08	0,20	19,42	0,10	26,71	0,12	27,17	0,06	26,85	0,03	30,52	0,06	28,40	0,08	32,23	0,12	28,35	0,07	25,23	0,17
**25**	19,89	0,20	26,79	0,19	19,09	0,19	26,02	0,19	26,11	0,10	25,05	0,14	28,47	0,09	26,49	0,17	30,69	0,20	26,23	0,08	21,90	0,12
**26**	20,14	0,13	26,50	0,09	19,03	0,10	25,97	0,03	26,42	0,25	24,90	0,16	27,56	0,14	26,48	0,08	30,15	0,36	26,60	0,26	22,32	0,12
**27**	19,97	0,14	27,95	0,31	19,45	0,10	26,22	0,33	26,28	0,07	26,33	0,04	29,27	0,25	27,56	0,09	32,06	0,09	28,21	0,26	25,37	0,14
**28**	20,09	0,05	28,26	0,10	19,15	0,15	26,91	0,24	26,96	0,09	25,92	0,09	28,60	0,23	26,71	0,05	30,60	0,14	27,66	0,40	22,83	0,17
**29**	20,58	0,21	27,32	0,23	19,54	0,11	24,33	0,12	24,42	0,03	23,85	0,41	26,35	0,13	24,70	0,14	28,54	0,07	24,27	0,01	20,14	0,05
**30**	21,78	0,10	27,16	0,16	20,13	0,02	29,37	0,17	29,71	0,18	28,17	0,03	32,09	0,13	29,72	0,13	33,93	0,07	30,06	0,09	25,57	0,13
**31**	20,03	0,15	27,18	0,12	19,20	0,07	27,68	0,03	27,66	0,13	26,81	0,01	29,67	0,23	27,80	0,10	31,88	0,32	28,24	0,20	23,62	0,10
**32**	20,23	0,16	26,87	0,08	18,85	0,12	26,31	0,06	26,78	0,16	25,41	0,01	28,54	0,32	26,92	0,14	31,21	0,43	27,74	0,42	23,52	0,16
**33**	19,68	0,20	26,79	0,36	18,77	0,09	27,72	0,20	28,58	0,07	27,43	0,02	30,78	0,21	28,80	0,05	32,47	0,17	28,64	0,45	24,53	0,13
**34**	20,67	0,19	26,64	0,06	19,28	0,14	26,23	0,26	25,03	0,17	24,39	0,14	28,01	0,44	25,87	0,20	30,69	0,35	24,12	0,35	22,19	0,11
**35**	20,14	0,08	28,47	0,12	19,47	0,08	28,99	0,20	29,51	0,14	27,76	0,09	31,92	0,36	29,33	0,16	33,31	0,17	30,03	0,15	25,89	0,10
**36**	20,16	0,10	26,65	0,35	18,96	0,20	25,75	0,02	26,02	0,10	25,15	0,08	28,07	0,16	26,48	0,14	30,25	0,17	26,47	0,14	22,28	0,09
**37**	19,51	0,07	28,08	0,40	19,24	0,11	28,20	0,40	28,37	0,18	28,30	0,13	31,32	0,20	29,37	0,08	32,86	0,04	29,09	0,04	24,83	0,22
**38**	19,43	0,12	27,92	0,17	19,35	0,08	26,91	0,23	26,51	0,18	26,24	0,11	28,95	0,12	27,75	0,07	32,02	0,13	27,41	0,15	23,79	0,13
**39**	20,10	0,11	28,62	0,15	19,87	0,18	24,44	0,10	23,32	0,14	23,35	0,05	25,93	0,13	24,18	0,15	28,31	0,21	24,41	0,16	20,26	0,13
**40**	20,61	0,23	28,63	0,25	19,41	0,26	25,44	0,42	24,97	0,13	24,40	0,61	27,00	0,62	25,04	0,21	28,96	0,31	25,77	0,36	21,79	0,36

For Cq SD, a value of 0,00 indicates either that no signal was detected in the triplicate (in this case mean Cq = N/A), or that a signal was detected only in one well of the triplicate (mean Cq calculated from a single value). In both cases or when mean Cq was ≥ 38, target gene was considered as no expressed.
