# Supplementary figures and images for: Use of a RT-qPCR Method to Estimate Mycorrhization Intensity and Symbiosis Vitality in Grapevine Plants Inoculated with Rhizophagus irregularis
Source: Plants (Basel). 2022 Nov 25;11(23):3237. doi: 10.3390/plants11233237 (PMC9741363; doi:10.3390/plants11233237)

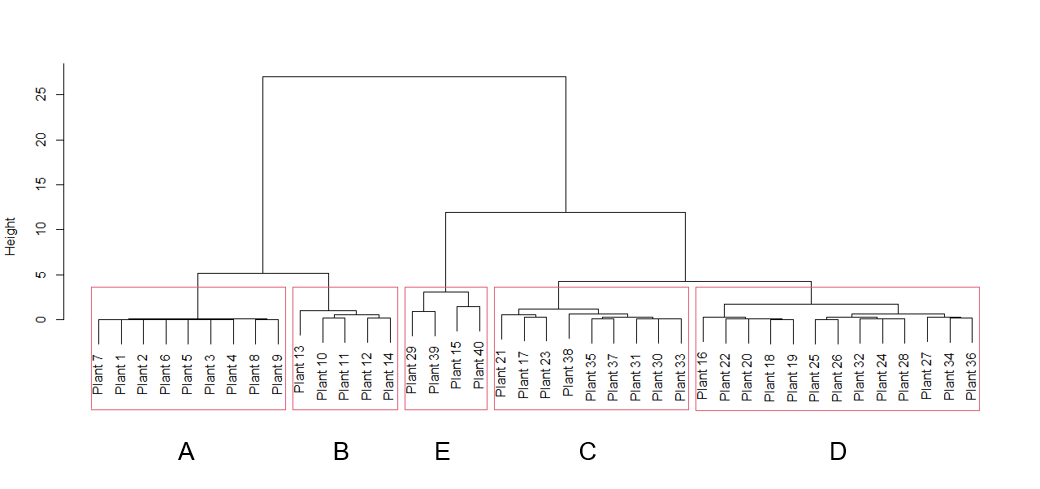

Supplement: Supplementary file 1 [file plants-11-03237-s001.zip › Supplemental-figureS1.tif]

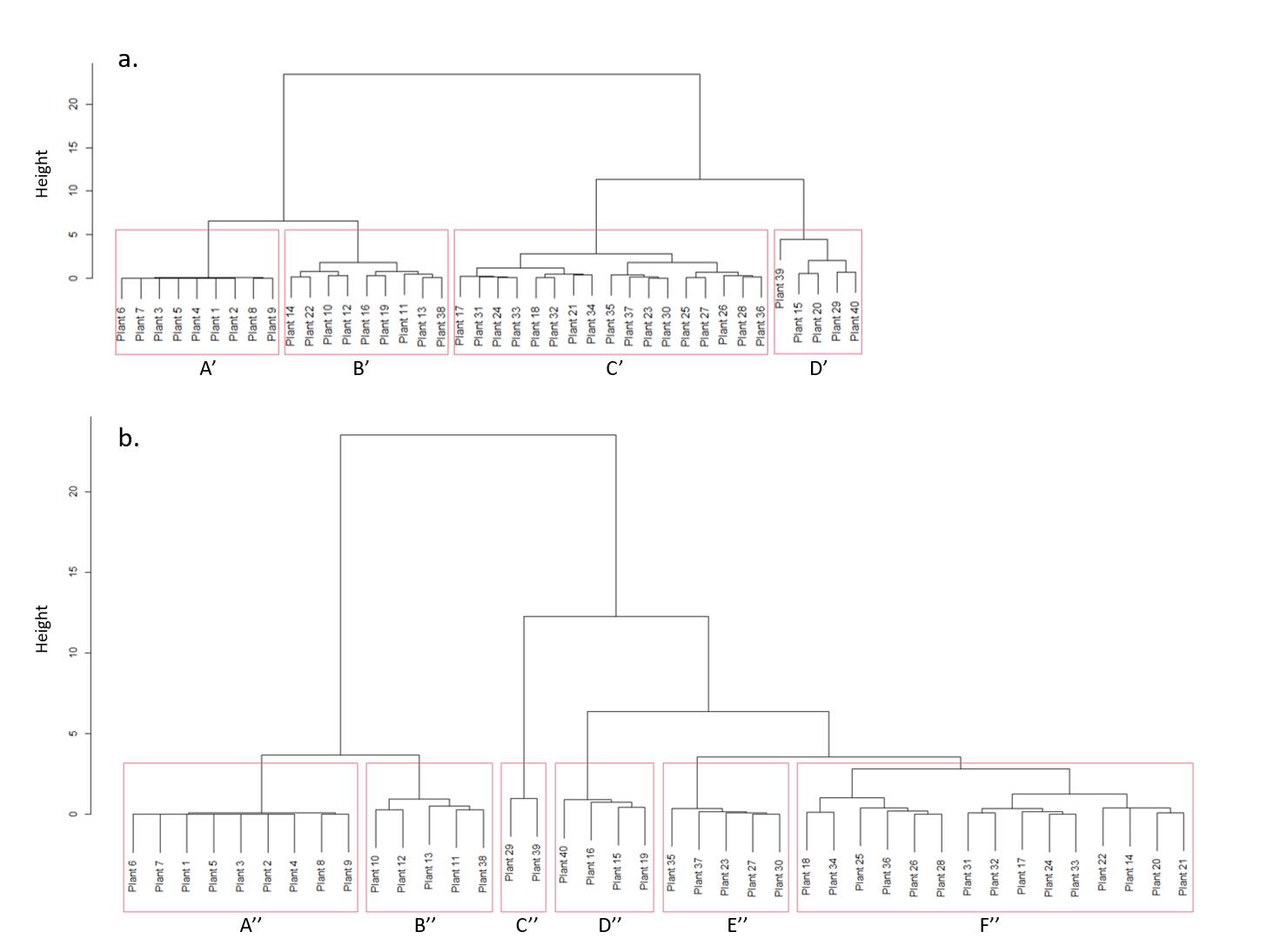

Supplement: Supplementary file 1 [file plants-11-03237-s001.zip › Supplemental-figureS2.tif]
